# Supplementary material for: Disruption of LLM9428/OsCATC Represses Starch Metabolism and Confers Enhanced Blast Resistance in Rice
Source: Int J Mol Sci. 2022 Mar 30;23(7):3827. doi: 10.3390/ijms23073827 (PMC8998287; doi:10.3390/ijms23073827)

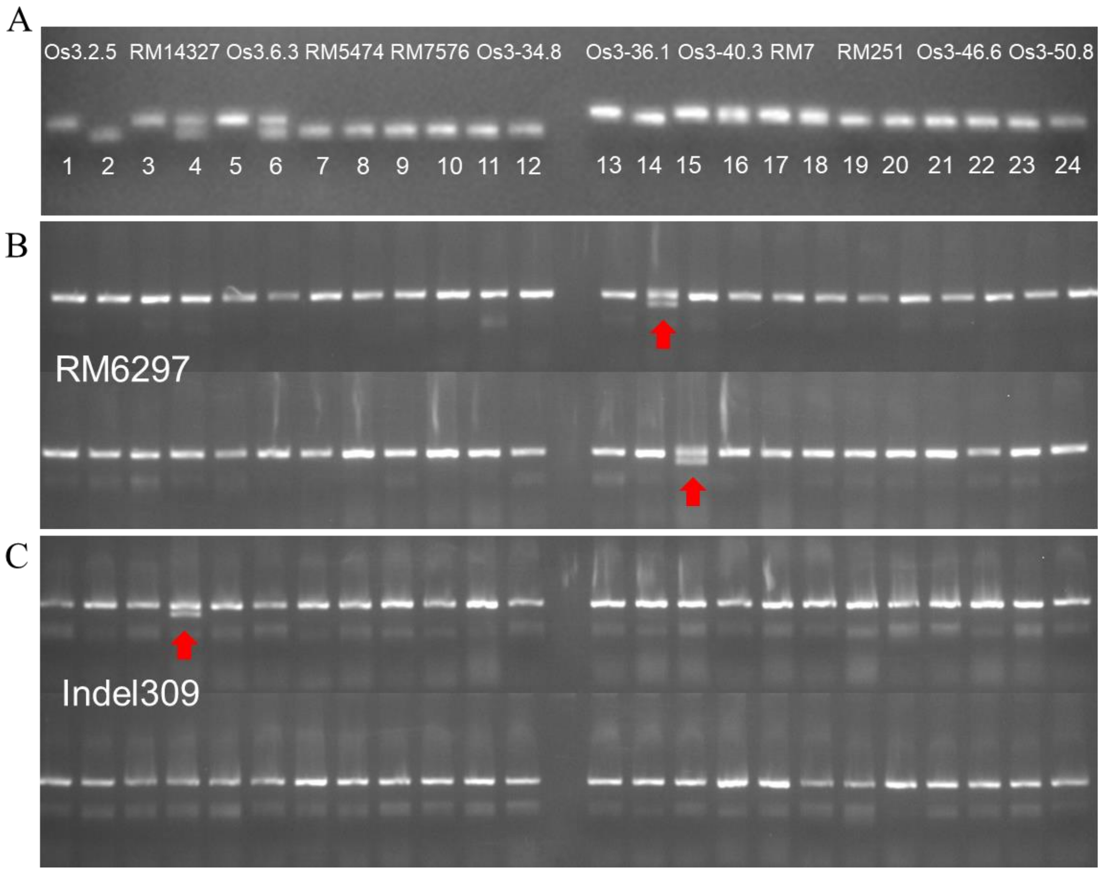

Figure S1: SSR marker analysis for Primary and fine mapping of *LLM9428*

(A) Primary gene mapping of *LLM9428* between SSR markers RM5474 and Os. 3.6.3. Odd number (1, 3, 5, …) refers to the plants without lesions phenotype (dominant plants) and even number (2, 4, 6, …) refers to the plants with lesions phenotype (recessive plants). (**B**) The molecular marker RM6297 showing polymorphic bands in F_2 recessive plants. (**C**) The molecular marker Indel309 showing polymorphism in F_2 recessive plants.

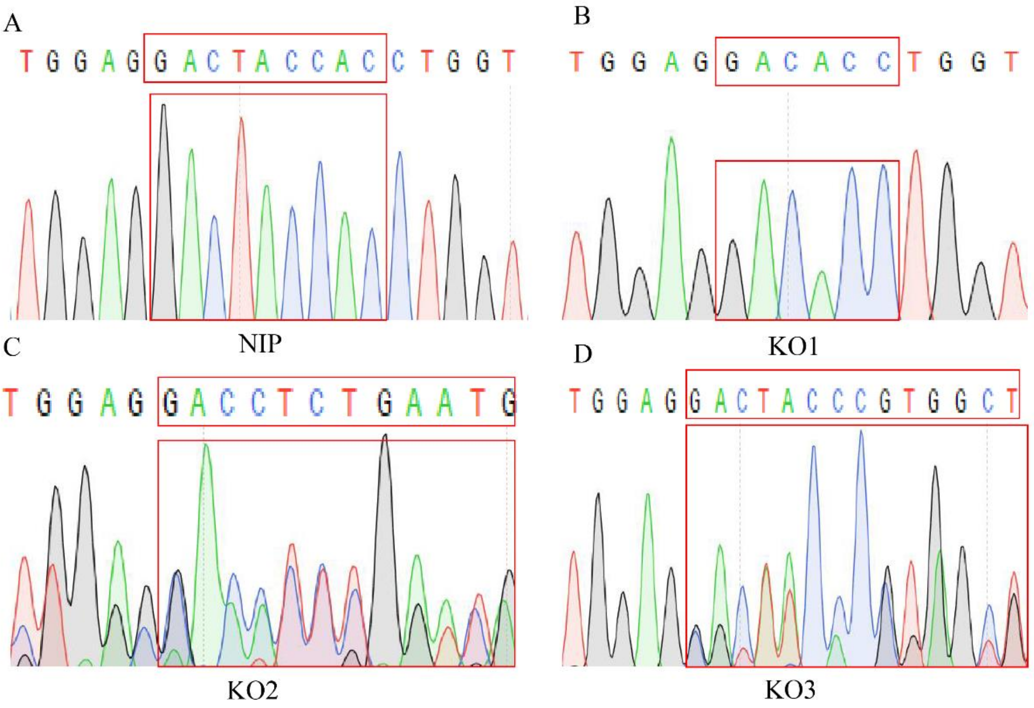

Figure S2: Chromatograms of NIP and CRISPR-KO lines

(A) Chromatogram of NIP showing the sequence of *LLM9428*, **(B)** Chromatogram of KO1 showing the sequence of *LLM9428*, **(C)** Chromatogram of KO2 showing the sequence of *LLM9428*, **(D)** Chromatogram of KO3 showing the sequence of *LLM9428*.

Table S1. List of primers used in quantitative real-time PCR.

Primer	Forward primer	Reverse primer
ACTIN	ACCATTGGTGCTGAGCGTTT	CGCAGCTTCCATTCCTATGAA
LLM9428	TTGCCAAGGAGAACAACTTCAAGC	TGATGAACCGGTCTTGCCTTGC
PR1a	CGTGTCGGCGTGGGTGT	GGCGAGTAGTTGCAGGTGATG
PR1b	TACGCCAGCCAGAGGAGC	GCCGAACCCCAGAAGAGG
PR10	AAGTTGCAGGTGGGGGATA	CTCAATGGCGTCGATGAAG
OsSSI	TATAAGGCCCGATGTGCCTCTG	GTCACCAGATCCAAGCATGACG
OsSSIIb	TGGAGATGCAGGTGCAGTAAGC	CCACAACATCTCCAAGACCACCTG
OsBEIIb	TTGCACCAAGTAGTCGTTTCGG	GGTATTATTTGACGCATGGCTGTG
OsAGPL1	ATGTGCTCCTGTTGGAGAGAGTCG	TACACGGCCTGAACTGTCGAAC
OsAGPL3	ACTGCTGGCTGAAGGAAAGGTTC	TATCTGCTTCCTGTACACCCTCAG
OsTPT1	TGTCCTTGGACAGCAAGTTCCC	TGATGCCATTGAAACACCAAGCAC
OsTPT2	CATTTGCCCACACCATTAAAGCTC	TGCTGGCCAAGGATAAACTGTG
OsSUT2	ACCATATGCAATGGCTGCTAGTCG	ACCCAGTGACACAATAACCTGTGG
OsSUT4	AACAGGAGGTGGACAAGGTCTG	CTGCGGAACAACGATTGCAAGG

Table S2. List of primers used as SSR and Indel markers.

Primer	Forward primer	Reverse primer
Os3.2.5	GGTTATGATTCGTTGGAAAA	AAACAAATACTCCCTCTCAAAA
RM14327	GATGCAGTAGGAACACCAAACAGC	ATCGAGTACCAAGTGCCTGTGC
Os3.6.3	TCATGTCCTCACTTTAACCC	CTCTTGAATCCCTCTCACAG
Os3.11.5	ACATGTCATCTTCTCTCTCGCTACC	GGAGACCACTCGTCACTTCG
RM5474	GTGGGTTTGTGTTTGGAGAGACG	GTGTTGGTGAGCATAGCAGTTGG
RM7576	CTGCCCTGCCTTTTGTACAC	GCGAGCATTCTTTCTTCCAC
Os3-34.8	TCTTGCAGTGTTCTTCAATC	GACTCCTTCTCGCCTACTTA
Os3-36.1	CTCCTTCCTCTCGTCCTC	CAACGGCATAACACCATT
Os3_40.3	ACAAGCATCCCTATGAAAGA	ATCACCGATATGAGCAATTC
RM7(3-9)	TTCGCCATGAAGTCTCTCG	CCTCCCATCATTTCGTTGTT
RM251	GAATGGCAATGGCGCTAG	ATGCGGTTCAAGATTCGATC
Os3-46.6	ACATCGAGTTTAATTGGCAT	AGAGAGAAAAGGTAGGTGGG
Os3-50.8	TTTGGAGTTAGTAGTGGGCT	CGAGTTCTTCCTGATCTGTT
RM6297	CTCTCCTCTCGCTCGCTGTTCC	GAGTGGTAATTGGGCTTGGATGG
RM14315	AACGGTAGCAATACGTTGATGG	GATTGTGGTCATTTGCTTGC
RM14360	CTCCTTGGATTTGTGCTTGTGC	CCTATAGCCCTCCTCTCCTTTGC
Indel-303	TGTGTGAGATGTTTCCCTGTTC	CGTGGCACAACAAATTGAGA
Indel-304	AGAAGGGGTCGGTTGAGTTT	TCTAACGGATAGCGGTGGAC
Indel-306	GGGGCGATATTCTACCATCA	TTCAGGCACTTTAGCTGCAA
Indel-309	GCAATGTGATATTTGTACAGAAAAGTG	TGCTTATCGGTTCTGACAGC
Indel-312	ACGGACCAGCAGAATACACC	AAGGCCGAGACCGAATTTAT
Indel-313	CATGATTCTGCAATTTATCAAC	GGTGCTGGTGCAGGTAAC
Indel-319	GTATACTACCCGAATTGTCACG	GATCTGTGAGTTTGGGATGG

Table S3. Genetic linkage map of chromosome 3 markers, primer, physical location and genetic distance

Marker No.	Primer	Physical location (Japonica database cv.02428)	Genetic Distance (cM)
ori			0
3-1	os3-2.5	890110	3.2
3-2	RM14327	1507052	5.4
3-3	os3-6.3	1523578	5.4
3-4	RM5474	3804260	13.6
3-5	RM7576	6079402	21.7
3-6	os3-34.8	6961942	24.9
3-7	os3-36.1	7050732	25.2
3-8	Os3_40.3	8298093	29.6
3-9	RM7	9829641	35.1
3-10	RM251	9949856	35.5
3-11	os3-46.6	10117095	36.1
3-13	RM5748	12327987	44.0
3-14	Os3_65.4	14473781	51.7
3-15	RM3204	14992775	53.5
3-16	RM7642	18632120	66.5
3-17	RM15281	18682472	66.7
3-18	RM3646	21997941	78.6
3-19	RM16	23127725	82.6
3-20	RM5626	24866396	88.8
3-12	os3-50.8	25591646	91.4
3-22	RM15721	25822460	92.2
3-21	RM426	27595690	98.6
3-23	RM8277	28811832	102.9
3-24	Os3_130.7	29437392	105.1
3-25	Os3_135.7	30066189	107.4
3-26	os3-136.5	30272881	108.1
3-27	RM3199	30422335	108.7
3-28	os3-142.3	31755536	113.4
3-29	RM143	33191366	118.5
3-31	RM422	33716593	120.4
3-30	RM3684	34623406	123.7
3-32	RM7389	36162247	129.2
3-33	RM85	36348226	129.8
end	end	36413819	130.0

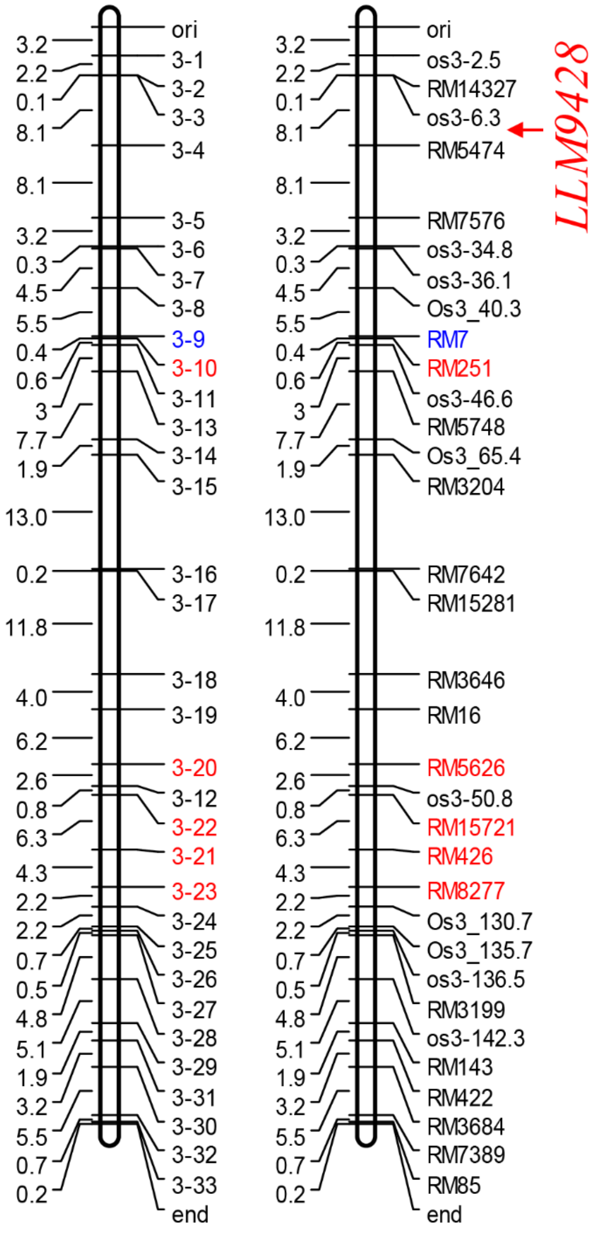

Supplement: Supplementary file 1 [file ijms-23-03827-s001.zip › ijms-1632305-supplementary.pdf]
